# Leukaemia mortality among young people in growing French communes.

**DOI:** 10.1038/bjc.1994.273

**Published:** 1994-07

**Authors:** L. J. Kinlen


*Br. J. Cancer* (1994), **70**, 180 © Macmillan Press Ltd., 1994

**LETTER TO THE EDITOR**

### Leukaemia mortality among young people in growing French communes

**Sir** – Laplanche and Vathaire (1994) report that no excess mortality from leukaemia among young people was found in French communes whose population had more than doubled. They contrast these observations with the significant increase in leukaemia in children under 5 in rural new towns in Britain during their rapid growth period (Kinlen *et al.*, 1990). Since no increase occurred in British overspill new towns, any comparison of the two studies requires that we know how closely the French communes in question approach the rural new towns studied in Britain.

Of the four British rural new towns that showed excesses of childhood leukaemia, three were well separated from population centres and based either on a small community (Glenrothes, population 1,100 rising more than 11-fold to 12,750 in just over 10 years) or on greenfield sites with a negligible population (Aycliffe, 60 rising 200-fold to 12,000; and Peterlee, 200 rising 65-fold to 13,000 in just over 10 years). The fact that all the communes were required to have an initial population of over 10,000 indicates that no such example was included in the French study. The fourth rural new town was an existing English town (Corby, population 13,000) into which many people from both industrial and rural Scotland moved so that in just over 10 years its population grew to 36,000. These facts resulted in mixing between people of a variety of origins, plausibly increasing the number of meetings between individuals who were both susceptible to and infected with the agents postulated to cause childhood leukaemia.

It would be useful for the interpretation of their study if the authors could present further details of the growth communes outside the 'Ile de France', giving details of leukaemia below age 5 for any that are comparable to the rural new towns in terms of extent of increase, type of incomers and proximity to population centres.

L.J. Kinlen
Department of Public Health & Primary Care,
University of Oxford,
The Radcliffe Infirmary,
Oxford OX2 6HE, UK.

**References**

KINLEN, L.J., CLARKE, K. & HUDSON, C. (1990). Evidence from population mixing in British new towns 1946–1985 of an infective basis for childhood leukaemia. *Lancet*, **ii**, 577–582

LAPLANCHE, A. & DE VATHAIRE, F. (1994). Leukaemia mortality in French communes (administrative units) with a large and rapid population increase. *Br. J. Cancer*, **69**, 110–113.


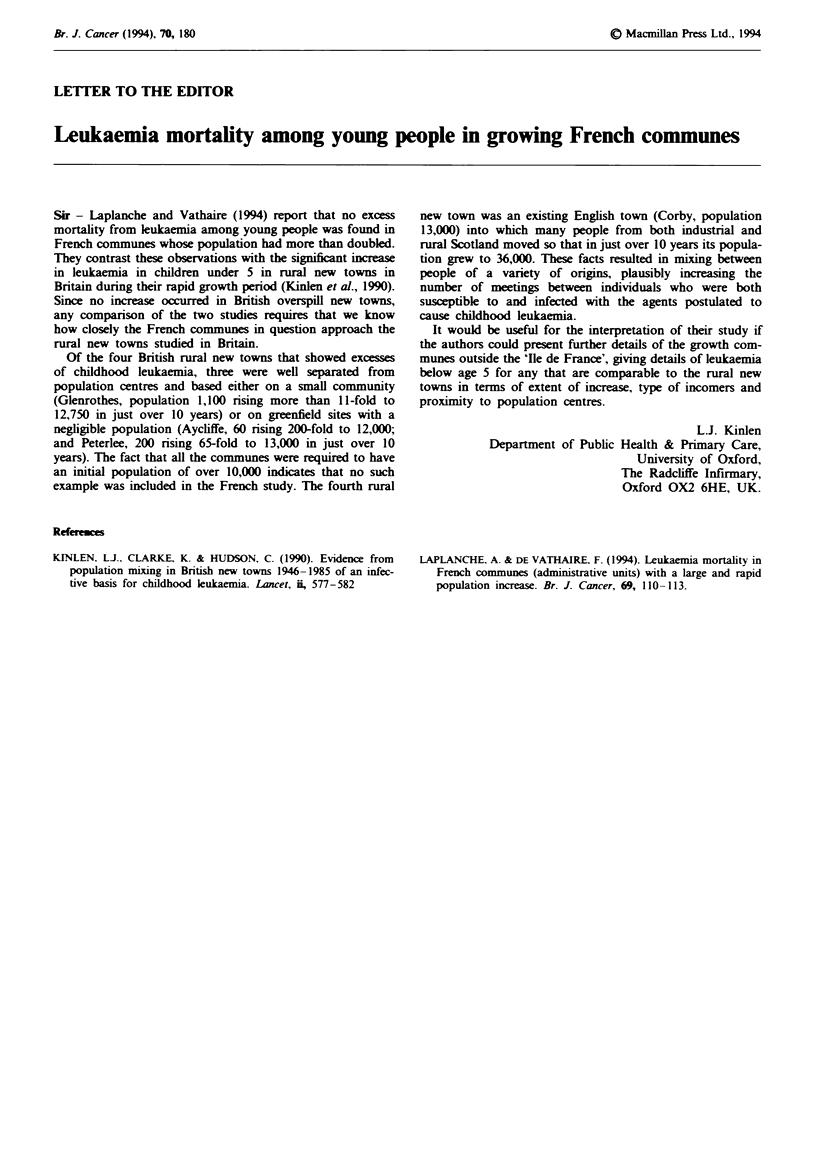


## References

[OCR_00054] Kinlen L. J., Clarke K., Hudson C. (1990). Evidence from population mixing in British New Towns 1946-85 of an infective basis for childhood leukaemia.. Lancet.

[OCR_00059] Laplanche A., de Vathaire F. (1994). Leukaemia mortality in French communes (administrative units) with a large and rapid population increase.. Br J Cancer.

